# The role of space-based observation in understanding and responding to active tectonics and earthquakes

**DOI:** 10.1038/ncomms13844

**Published:** 2016-12-22

**Authors:** J.R. Elliott, R.J. Walters, T.J. Wright

**Affiliations:** 1 COMET, Department of Earth Sciences, University of Oxford, Oxford OX1 3AN, UK; 2 COMET, School of Earth & Environment, University of Leeds, Leeds LS2 9JT, UK

## Abstract

The quantity and quality of satellite-geodetic measurements of tectonic deformation have increased dramatically over the past two decades improving our ability to observe active tectonic processes. We now routinely respond to earthquakes using satellites, mapping surface ruptures and estimating the distribution of slip on faults at depth for most continental earthquakes. Studies directly link earthquakes to their causative faults allowing us to calculate how resulting changes in crustal stress can influence future seismic hazard. This revolution in space-based observation is driving advances in models that can explain the time-dependent surface deformation and the long-term evolution of fault zones and tectonic landscapes.

## Abstract (web summary)

Earthquake prone areas are now routinely monitored by satellites, which can map surface rupture and distribution of slip on faults. Here Elliott *et al*. review the latest advances in the field of spacebased earthquake observations showing how this is used to understand active tectonic processes.

The study of active tectonics is primarily concerned with the deformation of the Earth's surface. This process results in the growth of mountains, rifting of continents and evolution of the geomorphic landscape. We aim to understand the material properties and processes that control the distribution of strain in the Earth's crust, from mobile belts to rigid cratons. An important consequence of the movement of the Earth's crust is that the slow accumulation of strain in the cold, brittle upper part of the crust (which builds up over hundreds to thousands of years) must eventually be released, often in earthquakes. Understanding the fundamental processes of tectonics will contribute to mitigating the growing risk of an increasingly urbanised population exposed to such hazards^1^.

Earthquakes occur when the accumulation of strain in the interseismic period results in a level of stress that can no longer be supported by friction on the fault^2^. The long-term accumulated elastic strain is recovered in the coseismic event resulting in a net translation of material either side of the fault. There are two important and measurable outcomes of this sudden strain release. First, the transient excitation of elastic waves from the energy released, which propagate through the Earth as body waves or along the free surface as surface waves and can result in damage to buildings. These waves can be measured by seismometers globally, and can be used very quickly (<30 min) to issue alerts on potential impact^3^. Second, the permanent displacement of the surface with respect to the far-field can be measured geodetically by methods such as triangulation and Global Navigation Satellite Systems (GNSS)^4^, and more recently by remote methods such as interferometric synthetic aperture radar (InSAR)^5^ and optically derived offsets^6^.

The recent flourishing of new satellite systems has made systematic, large-area observations possible, and our need to better understand active tectonics is increasingly pressing as populations in areas of high seismic hazard continue to grow and densify into urban centres (Fig. 1). Some of these megacities lie on top of or along-side major fault lines, many of which have not ruptured in historical times due to long earthquake recurrence intervals. Identifying where and how fast strain is accumulating is one of the key requirements for improving the assessment of seismic hazard and by combining this with geomorphic fault mapping, we will better identify active structures. By examining past earthquakes, we can also apply the insights gained regarding the nature of fault rupture and segmentation to other regions expected to be earthquake prone, but that have not yet been hit by a major earthquake since records began. The scientific response to major earthquakes now begins with the analysis of satellite imagery, as illustrated for the April 2015 Gorkha (Nepal) earthquake^7^,^8^.

Although satellite remote sensing of faulting and tectonics is a recent development relative to traditional field-based or seismological methods, rapid developments over the last 20 years have led to mature techniques and the adoption of standardised methods. However, we have often been limited in the past by access to sufficient quality and quantity of data, and a lack of systematic acquisitions from dedicated spacecraft. A new generation of satellites heralds a golden age for tectonic remote sensing, as earthquake seismology reached in the 1980–90s, and will enable the full potential of these techniques to be reached over the next two decades, complementing seismology, GNSS and field measurements. We anticipate this will lead to major advances in our fundamental understanding of earthquake hazard and tectonic processes. Many of these remotely sensed observations are critical for the fundamental science goals of establishing the nature of faulting and slip in individual earthquakes, and estimating the subsequent modifications to seismic hazard following such events^9^.

Whilst the basic notion of the earthquake cycle is now over 100 years old^10^, only in the past two decades have satellite systems allowed estimates of crustal deformation rates on large spatial scales of 100–1,000s km^11^. We review these recent developments in satellite technology and the resulting advances that they have brought to the study of active tectonics. We discuss how satellite observations are used in the immediate response to earthquakes. For example, the International Charter on Space and Major Disasters provides a unified system of space data acquisition and delivery to those affected by natural or man-made disasters. We show how the data are used in the longer term to build on the scientific understanding of deformation processes to improve assessments of future seismic hazard. The current step change in the consistency and global coverage of new earth observing systems, along with a recent shift to open and free data access policies, offers the potential for great advances in our understanding of earthquake hazard and the tectonic processes that continually reshape our world.

## Satellite systems

There are two main types of satellite Earth Observation systems that are useful for active tectonics (Fig. 2 and Box 1): those that actively illuminate the Earth's surface with their own radiation energy (mostly microwave radar systems in the form of SAR); and those that passively use the Sun's energy reflected off the Earth's surface or measure thermal infrared energy emitted from the Earth (optical/spectral satellites)^12^. These systems are usually in sun-synchronous low-earth orbits, typically at 600–800 km altitude with periods of 95–100 min. It is not possible to be exhaustive in covering all current and recent satellite platforms here^13^, and we instead focus on the main, successful civilian systems that provide regular, systematic and global or near global land coverage from a polar orbit. Additionally, we describe only microwave systems that have interferometric capability^14^ (InSAR) or optical data of relatively high resolution (<50 m) and that are used for the scientific study of active tectonics and earthquake deformation.

Developments in SAR and optical satellite technology have been concurrent with developments in our ability to use them for measuring deformation with InSAR^14^, constructing digital topography of active fault geomorphology to read tectonic signatures in the landscape^15^, and modelling these processes to understand the mechanisms of crustal deformation^16^. These latest technologies will also allow the realization of the ambition for useful data ‘Everywhere, All of the Time' to enable us to probe the deeper parts of fault systems, identify time-dependent deformation and transients, and ultimately connect reversible deformation that occurs on time scales of the order of earthquake recurrence to the long-term permanent growth of geological structures.

SAR systems have also been used to generate global topographic digital elevation models (DEMs). Although not satellite based, the Shuttle Radar Topographic Mission in 2000 produced near-global (56° S–60° N) ∼30 m, freely available DEMs over land. More recently, the TanDEM-X mission^17^, consisting of a pair of X-band SAR satellites, produced a global, commercial DEM for the Earth at 12 m resolution. As with SAR systems, it is possible to generate DEMs from optical imagery. An ASTER-derived near-global DEM at 30 m is freely available (GDEM^18^), as is another at 30 m (AW3D30) derived from the 2.5 m resolution PRISM instrument on ALOS-1 (ref. 19), with a 5 m version commercially available. It is also possible to generate bespoke DEMs from stereo satellite images^15^, which has been important for mapping earthquake ruptures with a quality approaching that of airborne light detection and ranging.

Scientific data access is a major issue for many satellite systems. When access is restricted or priced out of the reach of scientific studies, the use of data for long-term repeated measurements of deformation over large regions becomes impossible with such satellites. In contrast, the European Space Agency's (ESA) Sentinel-1 constellation has an open data policy, which will ensure that this is the main tool for measuring crustal deformation at small magnitudes over large areas for the next two decades. With the launch of Sentinel-1B (identical to 1A) in 2016, revisit periods of 6 days will now be possible, a great improvement on the earlier ENVISAT mission that had at best a 35 day interval. This is a significant advancement because short-period interferograms better maintain coherence and can capture time-dependent deformation, as well as provide a greater number of sampling observations to reduce atmospheric noise in measuring small strain accumulation.

The launch of a large number of satellites with the ability to observe the earth at optical and radar wavelengths, the shift to orbiting constellations of many identical or similar satellites (with some commitments by space agencies to maintain continuity of these systems over multiple decades), and the expansion in the commercial use of optical platforms at ever-increasing resolution have all occurred in the past few years. These systems are just beginning to be systematically and routinely exploited to respond to earthquakes and characterize the long-term hazard.

## Responding to earthquakes

Whilst we focus here entirely on satellite Earth Observation platforms for providing wide-scale data with global reach, there are strong complementary links with seismology, GNSS, such as the US Global Positioning System), fieldwork and aerial mapping, and geochemical dating of landscape features. In order both to fully constrain and advance understanding of many tectonic problems, it is often necessary to combine the suite of techniques listed above. Seismological observations are global in coverage for moderate and larger earthquakes and can provide the high-resolution temporal sampling necessary to look at the rapid evolution of an earthquake rupture over seconds, which is not possible using static measurements made from relatively infrequent satellite passes^20^. Seismology has been the principal tool for the study of earthquakes for most of the 20th century, particularly in remote areas where field observations are not easily made. The Worldwide Standard Seismograph Network was installed in the early 1960s and allowed for the routine determination of earthquake locations, mechanisms and magnitudes^21^. Observations from the Worldwide Standard Seismograph Network made an important contribution to our current view of plate tectonics, as the distribution of earthquakes were found to be generally tightly clustered, delineating non-deforming areas, particularly in the oceans. GNSS measurements yield a global scale model of present-day plate motions and have provided a global model of crustal deformation^22^, albeit with a relatively coarse spatial resolution for most parts of the world. Field and aerial observations can provide much greater ground resolution of ruptures and fold structures on local scales, which can be used to verify satellite observations; field observations can be used to study the longer term evolution of fault zones and tectonic landscapes over multiple earthquake cycles by dating features in the landscape that have been modified by tectonic processes.

## Mapping the disaster

**Figure 1:**
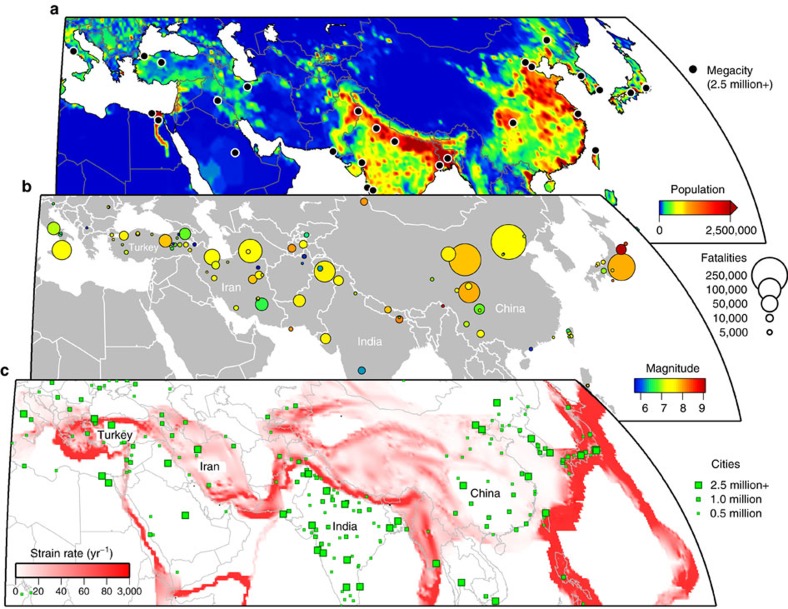
Distribution of current population relative to past fatal earthquakes and crustal strain across Eurasia. (**a**) Population count on a half-degree by half-degree grid for 2005. Megacities with populations over 2.5 million are marked by black circles. Data are the UN-adjusted population count from the Center for International Earth Science Information Network (CIESIN), Columbia University, United Nations Food and Agriculture Programme (FAO) and Centro Internacional de Agricultura Tropical (CIAT) (http://sedac.ciesin.columbia.edu/gpw). (**b**) Locations of past earthquakes in the period 1900–2015 resulting in more than 1,000 fatalities are denoted by circles coloured by magnitude and scaled in size by the number of fatalities (source: USGS, http://earthquake.usgs.gov/earthquakes/world/world_deaths.php). On the continents, faulting and earthquakes are more distributed than for the oceans. (**c**) Global Strain Rate Model (v2.1) showing the second invariant of the strain rate tensor^22^. This model is based on measurements from over 22,000 GNSS sites around the world. However, distributed over such a wide area, the resulting strain rate map is of relatively low spatial resolution for most of the globe. Large cities are overlayed (green) and scaled by population size.

**Figure 2:**
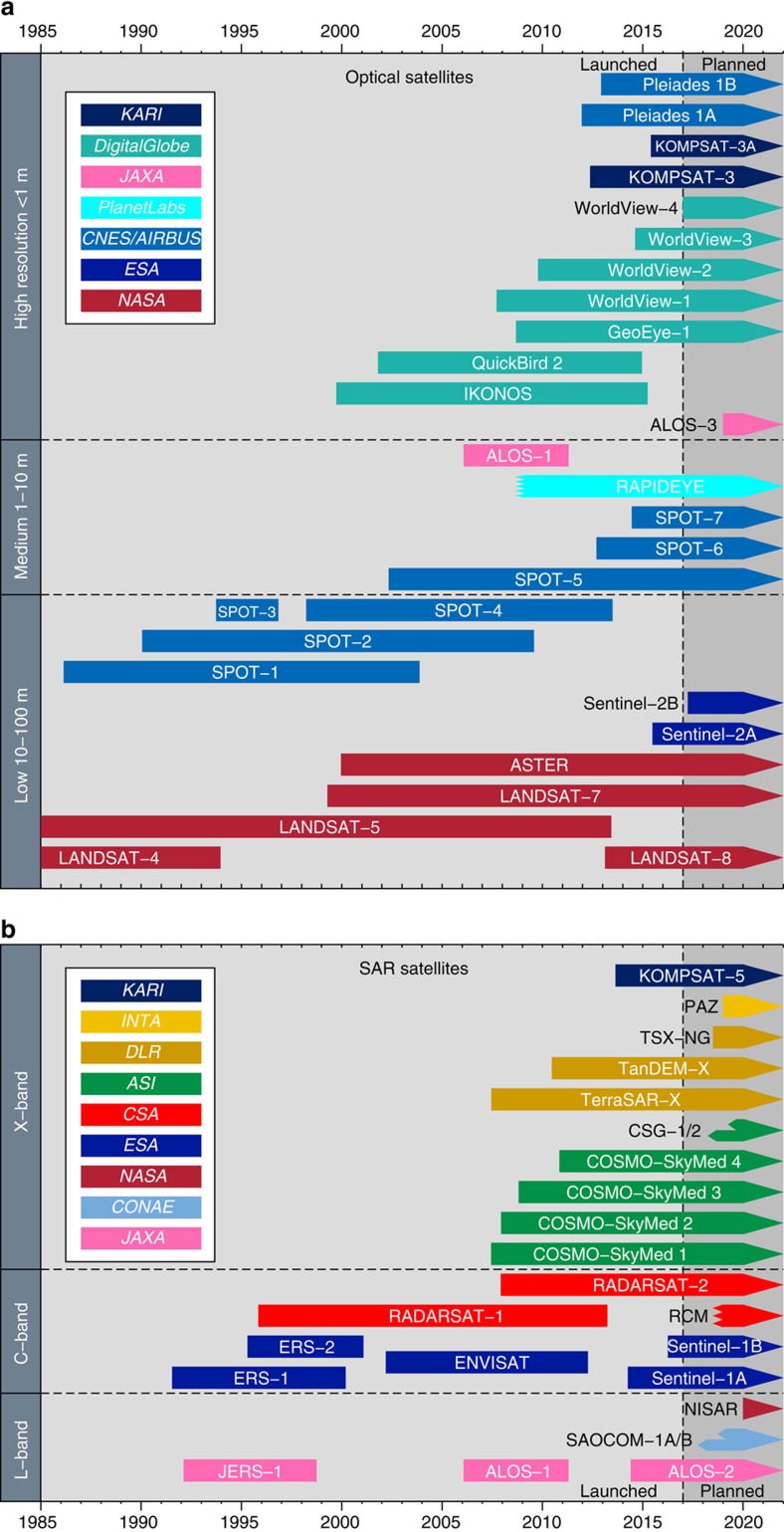
Optical and SAR satellite timeline for major Earth Observing satellites with systematic and global coverage. The history of satellite platforms in orbit from launch to instrument failure/de-orbit are shown for (**a**) the major optical satellites and (**b**) Synthetic Aperture Radars that have acquired images on a global basis, as of late 2016, coloured by operator. Also shown are anticipated upcoming launches, which are approximate and subject to change. Many other satellite systems exist for imaging the Earth^13^, but are limited in coverage, availability and suitability of the data for use in studies of earthquake deformation, and so have been omitted for brevity. Earth observing satellites before 1985 have also been omitted for clarity. SAR satellites are grouped by radar wavelength (L-band 15–30 cm, C-band 3.75–7.5 cm and X-band 2.4–3.75 cm^12^) and the optical satellites by the image spatial resolution. Note that the ERS-2 mission is shown as ending when gyroscope failure in February 2001 made interferometry almost impossible, but the satellite mission continued until September 2011. ALOS-1, as well as carrying a SAR, had a 2.5 m resolution panchromatic optical system with forward, backward and nadir looking cameras (PRISM), and a 10 m visible and near-infrared imager (AVNIR). Planned SAR missions with multiple satellites in a constellation are shown with split arrow tails. The acronyms for space agency operators or commercial owners are given in the legends. ASI, Agenzia Spaziale Italiana (Italian Space Agency); CNES, centre national d'etudes spatiales (France - National Centre for Space Studies); CONAE, Comisión Nacional de Actividades Espaciales (Argentina - National Space Activities Commission); CSA, Canadian Space Agency; DLR, Deutsches Zentrum fur Luft- und Raumfahrt (German Aerospace Centre); ESA, European Space Agency; INTA, Instituto Nacional de Técnica Aeroespacial (Spain - National Institute of Aerospace Technology); KARI, Korean Aerospace Research Institute; JAXA, Japan Aerospace Exploration Agency; NASA, National Aeronautics & Space Administration (USA).

Earthquakes present an increasing hazard to growing populations (Fig. 1). Satellite observations offer the potential for wide-area coverage (100–1,000 km scale) following a disaster; the International Charter on Space and Major Disasters^23^ is often invoked for the largest events^24^ to provide imagery quickly to governments and humanitarian agencies. Imagery collected in the aftermath of an earthquake can be useful for landslide mapping^25^, tsunami inundations in low-lying coastal areas^26^, building damage assessment^27^,^28^ and updates to seismic earthquake source models to improve estimates of ground shaking^29^. Recent advances have also been made in using coherence mapping with SAR satellites to look at building collapse and liquefaction^30^, which might not be readily visible in optical imagery when buildings undergo a pancake style collapse. There is however some latency in the use of satellite data for disaster response^31^, as it often takes several days or even weeks for satellites to image a certain part of the globe. This revisit capability is a particularly stringent limitation for InSAR, which requires suitable image acquisitions before and after the event in identical configurations^14^. The use of optical satellites is restricted to daytime, cloud-free conditions, which can cause difficulty, for example, for mapping landslides in mountainous regions. However, with an increasing number of satellites and also the existence of multi-satellite constellations such as COSMO-Skymed (Fig. 2), this latency is being reduced^32^, particularly for high-resolution optical satellites, which can have very flexible viewing geometries. The Disaster Monitoring Constellation^33^ is a multi-governmental Earth Observation resource, consisting of a series of medium to high-resolution optical satellites for rapid disaster mapping. This initiative has developed rapidly in the past decade and now boasts daily imaging capability at increasingly high resolution.

A scientific response to mapping earthquakes has a long history, with the 1906 California earthquake being the first to be studied geodetically^10^ from resurveying an established triangulation network, which led Reid to propose that the surface deformation either side of the fault was a result of the earthquake. Since the first use of InSAR to measure the coseismic deformation of the 1992 Landers, California earthquake^5^, the number of earthquakes studied with InSAR has exceeded 100 (ref. 11). InSAR data are able to provide remote measurements of subaerial coseismic ground displacements at very high-spatial resolution (∼10 m) from crustal (<50 km) earthquakes of moderate size (M_*w*_ 5+), limiting observations to continental earthquakes and only the largest subduction zone events that result in significant (>1 cm) on-shore deformation.

## Modelling the deformation

**Figure 3:**
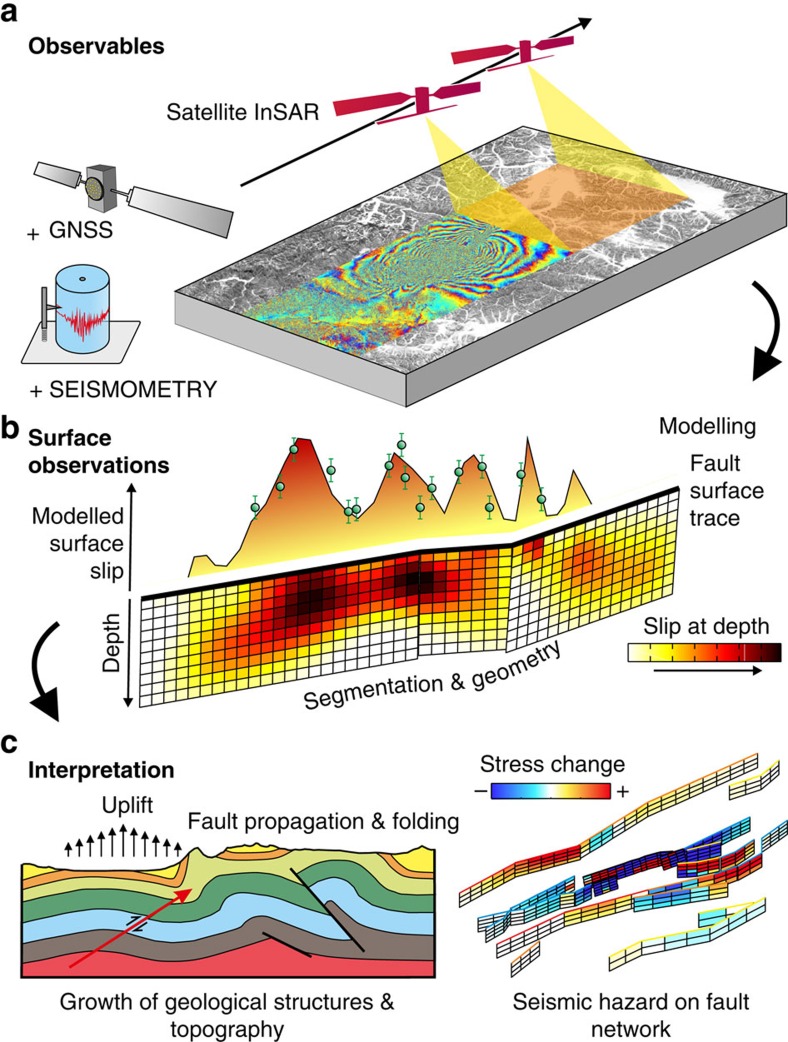
Work flow of observing and interpreting earthquake faulting and deformation using satellite data. (**a**) Earth observations are used to model and interpret earthquake ruptures and the geometries of faults and their slip distributions. Satellite interferometry provides high-resolution measurements of surface displacements by repeated illumination of the ground with radar over wide areas. This captures in its entirety the ground motion due to some of the largest continental earthquakes^7^,^8^. These observations can also be augmented with complementary data sets such as GNSS and seismometry to provide a time history of rupture^49^,^52^. (**b**) Based on these data and using elastic dislocation theory, it is possible to infer the slip across the fault at depth, as well as constrain the geometry and segmentation of faulting^41^,^42^. The modelled observations of slip in the near-surface can then be compared with field observations of discrete mapped surface offsets (green circles). Determining the geometry of faulting and its relationship with surface geomorphology is important for interpreting the surface fault expression^52^ and understanding the segmentation of rupture^20^ for estimating potential seismic hazard. Establishing the extent of slip is needed for determining which portion of the fault failed in the earthquake, and also which did not and could fail in future^8^,^41^. (**c**) By establishing the depth range of faulting and combining this with geological mapping and sections, it is possible to constrain the relationship between faulting and the growth of geological structures such as folds^119^ and topography^8^, as well as explore the potential control of lithology on both coseismic^119^ and postseismic slip^40^. Using the distribution of slip and fault geometry, it is possible to infer the changes of stress on the surrounding network of faults in an attempt to update the estimate of seismic hazard in a region^29^,^43^,^44^. Portions of faults which have undergone an positive change in stress will an increased seismic hazard, whilst those with negative stress changes will have been brought away from failure.

The aims of measuring earthquake displacement fields and modelling fault slip at depth (Fig. 3) are to improve understanding of earthquake mechanics and seismic hazard, and to deduce how the development and growth of geological structures relates to the deformation seen in individual earthquakes. Tectonic geodesy has enabled the development of models describing the evolution of slip over the earthquake cycle^34^, providing observations that can be used to relate the rheological properties in laboratory friction experiments to real-world fault systems^16^.

With the use of elastic dislocation theory^35^, it is possible to infer the geometry of the fault, the distribution of slip and to calculate the associated moment release. InSAR measurements are complementary to seismological observation of earthquakes: whilst they do not contain information on the slip history of a rupture, they provide much higher resolution spatial constraints on the fault slip patterns for shallow earthquakes, as well as being able to constrain more complicated fault segmentation, and are often jointly inverted with seismological data to solve for earthquake slip^36^. High-rate GNSS offers the potential to span this temporal divide by providing unsaturated dynamic offsets from passing surface waves as well as the permanent deformation^37^, although there are still only a few regions on Earth that have a GNSS network that is sufficiently widespread and dense enough for this technique. A disadvantage of InSAR is the repeat time of satellite passes, resulting in some postseismic deformation being captured with the coseismic signal^38^. This problem will be reduced with the shorter revisit times by an increasing number of radar satellite constellations (Box 1), but very rapid fault afterslip will still be incorporated into such measurements^39^,^40^.

As InSAR measurements image the displacement of the ground surface (relative to some assumed far-field undisturbed region), they are useful in determining the particular fault that ruptured at depth. This is not always obvious, as many major ruptures can be ‘blind', with the rupture not reaching the surface^8^,^41^,^42^. Determining the distribution of slip is important as it establishes which portions of the fault failed and which did not. From the distribution of slip, it is possible to calculate the stress transfer^43^ onto surrounding faults^29^,^44^ to identify regions of the fault system that have been brought closer to failure (Fig. 3). The sensitivity to discontinuities in the phase measurements present in InSAR data enables mapping of small displacements across other fault splays away from the main fault rupture^8^, allowing the identification of previously unmapped faults^45^,^46^ triggered by an earthquake.

## Imaging the fault rupture

**Figure 4:**
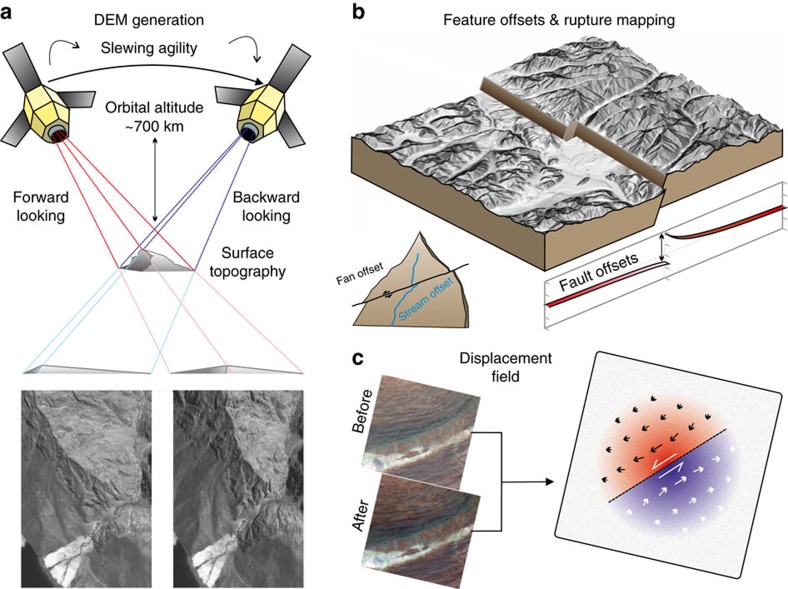
Derivation of DEMs from satellite optical data and examples of extraction of quantitative ground displacement data from the imagery and topography. (**a**) Information on the Earth's topography is extracted from pairs of satellite images through the derivation of a DEM. This technique makes use of the stereoscopic effect achieved from imaging the ground from two distinct positions in orbit. Regions of relatively higher relief are shifted more between the two images, as shown in the example where the mountain ridge crest is displaced towards the edge of the backward-looking image and is more in the centre of the forward looking image. The latest satellite systems are very agile and are capable of taking multiple images along the same orbital track. This permits stereo and tri-stereo acquisitions such as with Pleaides 50 cm panchromatic imagery from which it is possible to derive a DEM of near light detection and ranging quality^15^. (**b**) Derived products based on the DEM can then be use to extract quantitative landscape and tectonic information. Hillshade and slope maps aid with interpretation of surface features, and pick out where fault ruptures reach the surface. By tracing the lateral shift in a fault surface trace from an earthquake break and combining this with the derived DEM it is possible to determine the changing fault dip along strike of a rupture^120^. Fault perpendicular profiles, as well as displaced features such as offset streams and alluvial fans, provide estimates of fault displacement and can be compared with slip models. They can also provide estimates of longer term fault slip rates if the offset features can be dated, as well as looking for characteristic earthquakes^99^. By differencing DEMs from before an earthquake with ones derived after, it is possible to determine the height change resulting from the coseismic deformation, highlighting the many active faults structures which may be involved in a rupture^121^. (**c**) The optical imagery itself can be used to estimate the horizontal displacement field using optical correlation techniques^48^,^49^ which are based on estimating the shift of features within the imagery due to motion of the ground. This can provide rapid estimates of a fault surface trace as well as the degree of off-fault deformation^54^. They can be combined with InSAR measurements to derive a three component displacement field and also used in fault slip modelling.

For larger and shallower earthquakes that result in decimetre motion of the Earth's surface, it is possible to derive horizontal displacement fields from optical and SAR offsets (Fig. 4). This is achieved by using a method of sub-pixel correlation^47^, and these horizontal displacement fields can also be used to measure longer term tectonic motion^48^. Using a pair of 15 m panchromatic LANDSAT 8 images, the entire 220 km-long rupture in the M_*w*_ 7.8 Baluchistan earthquake was captured in a single-image footprint^49^, with estimates of the far-field noise being ∼0.5 m.

Such remote measurements of surface motion often provide a useful constraint on the three components of the displacement field^50^ when Global Positioning System measurements are lacking, as InSAR measurements are typically most sensitive to vertical motion^51^, whilst the optical pixel tracking retrieves the horizontal components. Additionally, the displacement field is more complete in regions of high strain near fault ruptures, where the high phase rate in InSAR can result in the loss of data. Therefore optical offsets can provide a relatively sharp (100 m scale) trace of the fault rupture, as was achieved using 2.5 m panchromatic SPOT imagery for the 2010 El Mayor Cucapah earthquake^52^. It is similarly possible to achieve offset measurements from pixel tracking of SAR amplitude images^53^ to retrieve the azimuth and range displacements, but SAR pixel resolutions are usually lower, so the displacement measurements are correspondingly noisier.

The utility of such offset measurements in providing constraints on the earthquake are twofold – first, delineating the rupture provides an a priori constraint on the fault geometry and segmentation; second, the horizontal displacement field can be used as a constraint in the inversion for the distribution of slip at depth, as well as providing a comparison with field observations of rupture at the surface (Fig. 4).

With the increasing resolution of optical satellites (Box 1), future fault rupture mapping and the derivation of displacement fields should greatly improve, achieving sub-metre scale resolution in the very near future, which is currently only possible using aerial images for limited areas. Global systematic coverage will provide important constraints on the degree of fault offset localized at the rupture versus the amount of off-fault deformation that occurs^54^, as well as in mapping separate fault splays and the deformation between fault segments and step-overs. However, the potential for the latest satellites to achieve this is limited by non-systematic acquisition plans of the commercial satellites, which are typically tasked on demand, as well as by the expense of these very high-resolution satellite products. This means that suitable pre-earthquake imagery for a given earthquake is less likely to exist than for the Landsat and SPOT satellites, which make/made regular global acquisitions.

Ultimately, the aims of harnessing the coverage of the latest Earth Observation satellites are to better understand the seismotectonic processes of earthquake rupture to determine the seismic potential of faults, as well as to map building collapse and forecast secondary hazards such as landsliding in the immediate aftermath of an individual earthquake. The goal is to develop time-dependent seismic hazard assessment following earthquakes^55^ and to help constrain dynamic models of rupture^2^,^34^. To achieve these aims, it is necessary to make use of the near-continuous suite of observations that are possible from orbiting systems to build up a long time-series of crustal deformation.

## Characterizing large-scale deformation and long-term hazard

The availability of large volumes of high-resolution remotely sensed data has already fundamentally changed how the scientific community responds to major earthquakes. However Earth Observation data are now also beginning to play a crucial role in estimating and understanding large-scale deformation and long-term seismic hazard on the decadal to millennial scale.

Traditional approaches for estimating seismic hazard are based on forecasting the probability of future earthquakes from statistical analysis of historical and instrumental seismicity catalogues^56^. This approach is fundamentally limited in regions where the average recurrence interval for earthquakes exceeds the length of the historical record^57^, which is the case for most continental active tectonic regions worldwide. This observational bias commonly leads to the repeated revision of seismicity-based hazard maps to retrospectively increase the hazard in the immediate area around the last major earthquake^58^. This approach is by definition backward-looking, and in areas with long recurrence intervals, the next large earthquake is unlikely to be in the same location as the last major event.

## Measurements of geodetic strain

**Figure 5:**
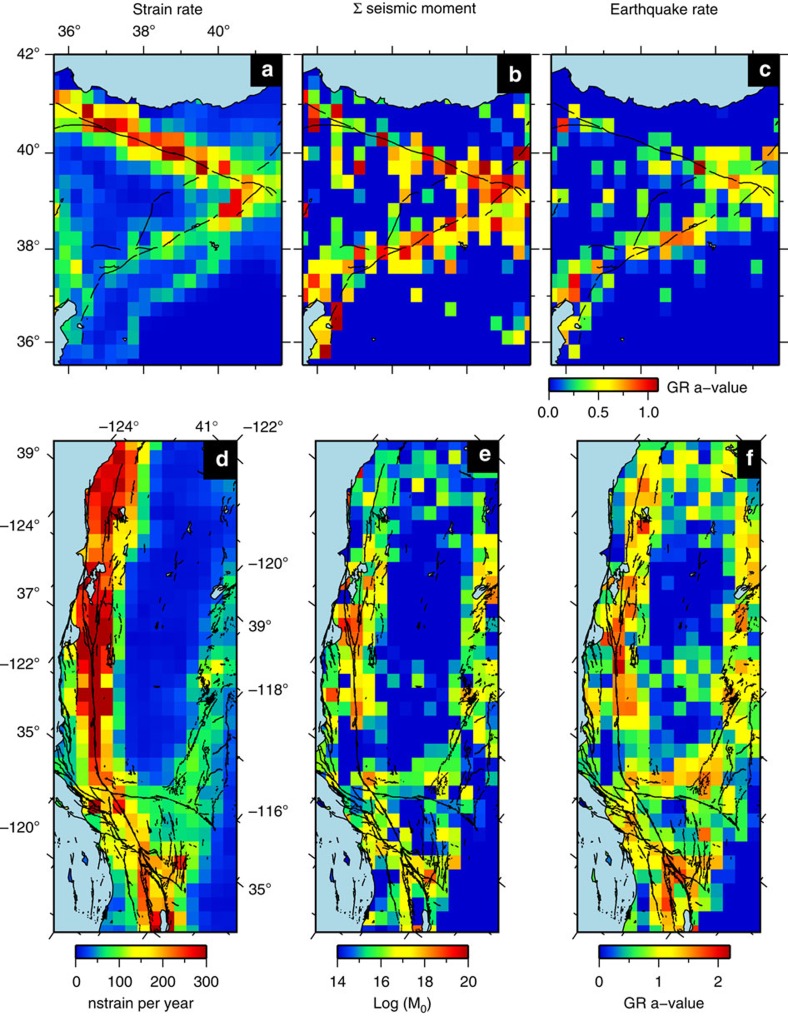
Comparison of strain rate against seismic strain and earthquake rate. (**a**) Second invariant of the horizontal strain-rate tensor from the Global Strain Rate Model v2.1 (ref. 22) for Eastern Turkey, showing localized strain on the North and East Anatolian Faults (NAF and EAF respectively). Active fault traces are denoted by black lines^122^. (**b**) The log of the summed seismic moment since 1000 AD, derived from the declustered SHARE historical and instrumental seismic catalogue^123^ and calculated at 0.25° resolution. (**c**) The Gutenberg-Richter *a*-value calculated at the same resolution from the same data. **d**–**f** show the same for California and the San Andreas Fault Zone, with seismic parameters derived from declustered UCERF3 seismic catalogue^124^. Fault traces show structures assumed active since the late Quaternary from the USGS (http://earthquake.usgs.gov/hazards/qfaults/). For both Eastern Turkey and California, there is a clear relationship between geodetic strain-rate and both seismic moment and earthquake rate.

**Figure 6:**
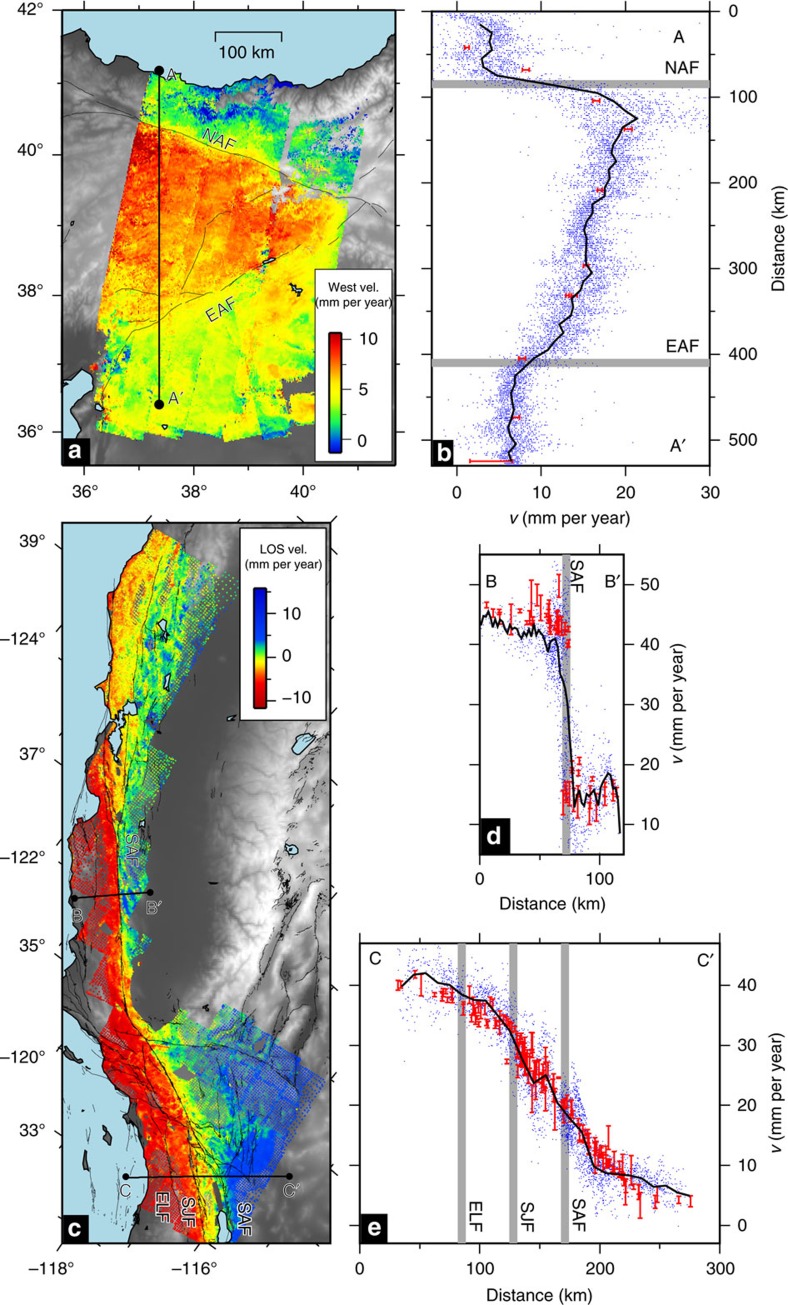
Examples of large-area interseismic measurements with InSAR. (**a**) InSAR interseismic crustal velocities in Eastern Turkey^79^. A mosaic of five overlapping descending and ascending tracks from the ENVISAT satellite is shown, augmented with unpublished data from one additional descending track. Velocities are presented as westward motion only, under the assumption of zero vertical differential motion across the region and small sensitivity of the instrument to north–south motion. Line C–C′ shows the location of the profile in **b** through the North Anatolian Fault (NAF) and East Anatolian Fault (EAF). Active fault traces are denoted by black lines^122^. (**b**) Profile of westwards velocity where blue points are InSAR measurements and red bars are GNSS velocities with one sigma uncertainties, within 30 km of the profile lines. The black line shows the mean InSAR velocity and the grey vertical bars show the locations of major strike-slip faults. Localized deformation across the NAF and EAF, with little deformation away from these fault zones. The gradient of velocity in Anatolia, the region between the NAF and EAF, does not reflect strain but the bulk horizontal rotation of Anatolia. (**c**) InSAR interseismic crustal velocities along the San Andreas Fault Zone (SAF), California^78^, shown at the same spatial scale as panel **a**. Velocities are shown in the satellite's line of sight and are a mosaic of 13 overlapping ascending tracks from the ALOS-1 satellite. Lines A–A′ and B–B′ show the location of the profiles in **d**,**e** through the San Andreas Fault (SAF), San Jacinto Fault (SJF) and Elsinore Fault (ELF). Fault traces show structures assumed active since the Late Quaternary from the USGS (http://earthquake.usgs.gov/hazards/qfaults/). (**d**,**e**) Profiles of assumed fault-parallel motion where blue points are InSAR measurements and red bars are GNSS velocities with one sigma uncertainties, within 30 km of the profile lines. The black line shows the mean InSAR velocity and the grey vertical bars show the locations of major strike-slip faults. (**d**) Fault creep across the SAF, represented by a sharp step in velocity across the fault. (**e**) Distributed shear across three major and closely spaced faults in the San Andreas Fault Zone, the ELF, SJF and SAF.

An alternative and independent approach to estimating seismic hazard involves instead using geodetic measurements of interseismic strain-rate as a proxy for hazard in regions which are deforming fast enough to be accurately measured above the noise inherent in the technique (limited largely by atmospheric conditions discussed below). The theory of elastic rebound^10^ forms the basis of this method by considering the elastic potential energy budget in the seismogenic crust, comparing the rate of elastic strain accumulation to the rate of strain release in earthquakes. Geodetically derived crustal velocity fields from the interseismic period can be used to calculate the crustal strain-rate tensor, which in general agrees in both orientation and magnitude with the time-averaged seismic moment rate tensor in regions that are large enough that short-term variations in seismicity rate are not important^59^,^60^ (Fig. 5). The geodetically derived strain-rate can therefore be directly related to seismic hazard via assumed magnitude-frequency relationships for earthquake size distributions^61^. The major benefits of this approach are that it does not rely on incomplete seismicity records; the approach is based on characterizing the physical cause of earthquakes, rather than the rate of occurrence of the effect, that is, the earthquakes themselves. For areas where we possess detailed seismicity records in addition to strain-rate data, the combination of information on both recent strain accumulation rate and magnitude of past strain release makes it possible to better constrain the elastic potential energy budget in the seismogenic crust, and therefore also the likelihood of future large earthquakes. Differential GNSS measurements have been used for the last ∼25 years^62^ to measure interseismic velocities with millimetre per year accuracy, but with a low spatial density of measurements; whilst a few tectonic regions globally have dense GNSS networks, the typical station spacing in deforming areas is 50 km (contrast Fig. 6b,d). InSAR was first used to measure interseismic deformation with high-spatial resolution (∼160 m) over the North Anatolian Fault in Eastern Turkey^63^, and has since been used to make over 25 measurements of interseismic strain on faults worldwide^11^.

The ability of InSAR to measure small ground displacements or velocities is limited by the magnitude of other sources of delay to the differential radar phase, the largest of which are uncertainties in precise satellite orbits^64^, ocean-tidal loading effects near coastlines^65^ and spatio-temporal variations of the atmosphere, including both the refractivity of the troposphere^66^ and the electron content of the ionosphere^67^. Tropospheric signals are commonly the largest source of noise, often being several orders of magnitude larger than the interseismic signals of interest^66^, and on similar spatial wavelengths (∼10–100s km), limiting the detection threshold for these signals. Tropospheric delays can be mitigated by corrections which rely on auxiliary data^68^,^69^ or empirical methods^70^,^71^, and by time-series and stacking techniques^72^,^73^, which rely on temporal averaging and spatio-temporal filtering to improve the signal-to-noise ratio of the data. Therefore the accuracy of interseismic velocity measurements made with InSAR is directly determined by the development of noise correction and time-series techniques, and by the volume of data available for stacking and time-series analysis. In the past this has limited such measurements to faults with slip rates greater than ∼3 mm per year^74^.

Over the last decade, data volumes have increased rapidly due to a proliferation in the number of SAR satellites (Box 1 and Fig. 2) and significant advances have been made in the mitigation of sources of uncertainty inherent in InSAR data. This has lead to major improvements in our ability to retrieve long-wavelength and small magnitude interseismic velocity signals from SAR data sets. Modelling these high-resolution, wide-area and high accuracy maps of interseismic crustal velocities has led to correspondingly major advances in our understanding of many fundamental fault processes.

## Velocity fields and modelling of interseismic deformation

The first interseismic studies using InSAR measured interseismic crustal velocities over relatively small areas, from a single satellite look direction, and had large associated uncertainties due to contamination from non-tectonic signals. These studies used analytical elastic dislocation equations to model these data and estimate both fault slip-rate and the depth to which faults are locked by friction in the interseismic period^75^, with strong assumptions of a known slip direction imposed by the one-dimensional nature of these data. These simple elastic models have remained ubiquitous, with over 180 applications in the literature^11^, despite advances in our understanding of the complexity of the earthquake cycle. The continuing success of these models some 30–40 years after they were first suggested is partly due to their simplicity and effectiveness, but also due to the large uncertainties in geodetic interseismic data, which mean that until recently such data have been rarely able to robustly distinguish between these and more complex models. However, there is at times also a fundamental limit in the use of deformation solely at the Earth's surface in terms of resolving the kinematics of motion at depth, even in the face of vanishing measurement error, due to the surface equivalence of some deeper deformation processes^76^.

In contrast, modern studies that use large data volumes and advanced correction techniques to mitigate non-tectonic signals, now routinely measure interseismic velocities using multiple overlapping radar swaths with complementary satellite look directions. These studies commonly cover large areas spanning several hundred thousand square kilometres^77^,^78^,^79^,^80^, crossing entire plate boundary zones and multiple faults at high-spatial resolution, for example, in Eastern Turkey and the Western US (Fig. 6), with mm per yr level uncertainties.

Large-area, mm-accuracy InSAR data sets now offer a window onto interseismic fault processes and large-scale crustal deformation with unprecedented spatial scale and resolution. The derived velocity fields^77^ have been used to illuminate crustal deformation processes at the orogenic-scale, testing competing theories about whether the continental lithosphere deforms as a collection of discrete blocks or as a continuous medium^79^,^80^ and how mountain-belts grow in elevation during the interseismic period above large dip-slip faults^81^. They have also been used to investigate the spatial variation of rheology and frictional properties on faults, from the detection of heterogeneous rheology in the lithosphere, both at the large scale and in weaker localized regions around the fault^82^, to using spatial^83^ and spatio-temporal^84^ variations in aseismic creep on faults to infer spatial variations in dynamic frictional properties. These data can be used to map variations in frictional coupling on subduction megathrusts^85^, and to map structural complexity on continental faults at depth, showing how deep shear zone localization can differ from surface structural complexity and segmentation^86^, and highlighting deep crustal connections between branching faults^87^. This information is not only important for the fundamental understanding of earthquake and fault mechanics, but is also crucial for understanding both the likely propagation of ruptures in large earthquakes, and the proportion of plate-tectonic movement that is accommodated by earthquakes and by slow slip or creep.

Even with the use of overlapping radar swaths with multiple look directions, InSAR still has poor sensitivity to north–south crustal motion due to the polar orbiting direction^88^, which makes it more challenging to measure motion associated with north–south striking strike-slip faults and east–west striking dip-slip faults. The Multiple Aperture Interferometry/Spectral Diversity method^89^ allows the measurement of along-swath motion by using one standard pair of SAR images to create two interferograms that look forwards and backwards along the radar swath. The difference between these two interferograms yields the along-swath displacement, albeit with much lower precision than standard InSAR measurements of range change^90^. However, this method is nearly insensitive to atmospheric errors and it should be possible to measure interseismic velocities with this approach provided they vary slowly in space; if combined with standard InSAR it should be possible to obtain three-dimensional interseismic motion^91^. The application of InSAR to interseismic deformation suffers the same limitation as any application of surface geodetic data to subsurface problems – that of decreasing constraint on deformation processes with depth. This fundamental limitation of surface geodetic data has long been recognized^92^.

## Seismic hazard forecasting

The translation of interseismic strain-rates from geodesy into forecasts of seismic hazard is not straightforward but several different approaches have been developed and successfully tested^93^,^94^. Recently, global strain rates estimated from GNSS^22^ have been used to create a global forecast of seismicity^95^, and in future InSAR will be used to improve these forecasts in the many regions globally where GNSS site density is poor (for example, Fig. 6b). The major assumption necessary for the translation of geodetic to seismic strain is the proportion of geodetic strain that is released aseismically by slow slip, creep or plastic deformation, as opposed to in earthquakes. At present, such forecasts rely on calibration against seismicity records to determine how this factor varies across tectonic regions^95^, but it is likely that this information could be constrained in future by global high-resolution interseismic measurements from InSAR. Similarly, we envisage that other complications for the translation of geodetic to seismic strain will be resolved: strain-rate transients in geodetic data, fault interaction and fault structural control on dynamic rupture and earthquake magnitude will be better constrained in future by high-resolution InSAR data. However, faults with slip rates slower than 3 mm per year may require many years of observations for signals to emerge above InSAR noise levels. Whilst slowly deforming regions have a relatively low seismicity rate when compared with regions of faster deformation, it is important to note that large (for example, M_*w*_ 7.7 Bhuj, India 2001 & M_*w*_∼7.5 New Madrid, US 1812/13) and even great earthquakes (e.g. M_*w*_∼8.3 Shillong, India 1897 and M_*w*_ 8.0 Wenchuan, China 2008) can still occur in regions of low strain-rate such as the continental interiors, albeit with long recurrence intervals. The detection limits of geodetic techniques will improve with technological, methodological and data-driven advances, but seismic hazard in very low strain-rate regions, as well as in regions exposed to anthropogenically induced seismicity, will always be challenging to estimate using geodesy. Synoptic approaches to seismic hazard estimation that combine both seismicity records and strain-rate measurements have also been developed^96^, and can help overcome these limitations, and these approaches are likely to be more commonly adopted in future.

Earth Observation data also contribute to hazard estimates for faults on hundred-year, millennial and longer timescales, through the use of optical and multispectral satellite imagery for geomorphological analysis of active faults^97^. These data are used in three main ways, in addition to their use for the study of coseismic ruptures and displacement fields from modern and historical earthquakes. First, high-resolution optical and multispectral imagery and high-resolution DEMs can be used to identify active faults (Fig. 4), when they are interpreted within a framework for landscape evolution, based on the interaction between tectonic processes and erosion^98^. Second, these data sets can be used to identify and precisely measure offsets in geomorphic features and historical fault scarps for earthquakes over the Quaternary. By combining these offsets with geochronological tools for dating offset rock or sediments, for example, C^14^, Cl^36^, Be^10^ dating or optically stimulated luminescence, it is possible to obtain age ranges for previous earthquakes and/or an average slip-rate for the fault. With these techniques, it is possible to measure long-term average slip-rates with sub millimetre/year accuracy, significantly below the current threshold for geodetic techniques. These methods can therefore complement satellite-geodetic techniques by extending our ability to estimate seismic hazard to very low strain-rate regions. Third, these data can be used for the identification of repeated earthquake offsets, to characterize earthquake histories on faults where the slip in successive earthquakes has the same ‘characteristic' spatial distribution^99^. Recent advances in both the volume and resolution of satellite imagery, and its increased availability, have meant these techniques are widely used for seismic hazard identification and analysis on the country-wide scale; these methods complement geodetic techniques well due to the differing time scales.

## Implications for fault mechanics and how continents deform

The unprecedented explosion in the quantity and quality of space-based observations of the deforming planet have led to a rapid evolution in our understanding in a number of key areas of research, with further breakthroughs likely to arise as data sets improve over the next decade.

A key question is what controls the time-dependent deformation observed before, during and after earthquakes? Earthquake repeat times are much greater than the period for which we have good observations, so if we are to use decadal measurements of surface deformation to say anything useful about long-term hazard, we need to build models that can predict how deformation varies as a function of time and space around faults during the entire earthquake cycle.

**Figure 7:**
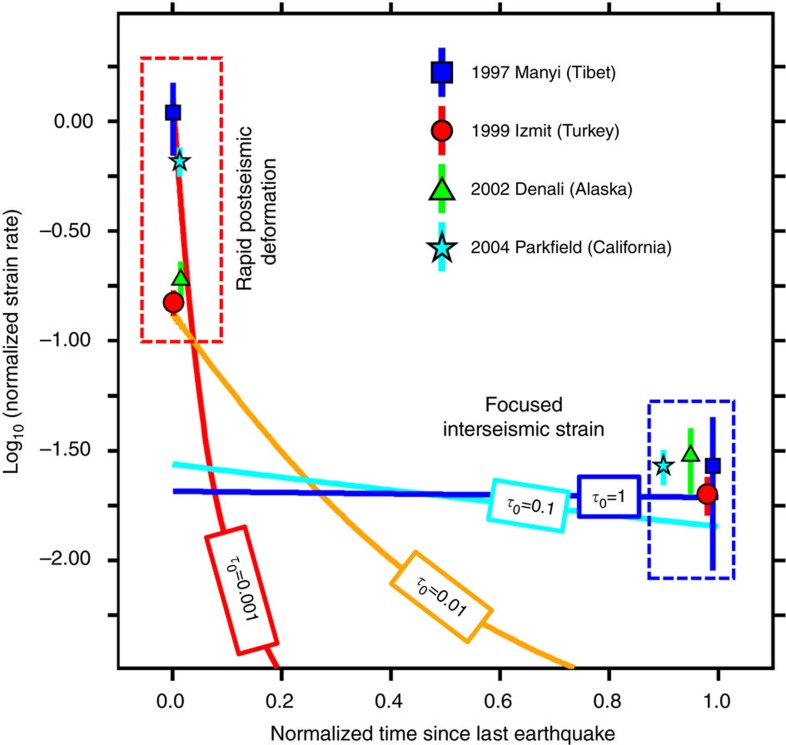
Time-dependent deformation at strike-slip faults. Interseismic and postseismic strain rates for four strike-slip faults where data are available before and after a major earthquake^74^,^105^,^108^,^125^,^126^,^127^,^128^. Strain rates in units of microstrain per year and are normalized by the long-term slip rate of the fault. Time is normalized by the average inter-event time for the fault. The curved lines show model predictions from a simple viscoelastic coupling model, with an average slip rate of 1 mm per year, in which repeating earthquakes occur above a viscoelastic substrate. The controlling non-dimensional parameter, *τ*_0_, is the ratio of Maxwell relaxation time to inter-event time; *τ*_0_ values of 0.001/0.01/0.1/1 correspond to a viscosities of ∼10^17^/10^18^/10^19^/10^20^ Pa s for typical inter-event times.

One approach has been to use the ergodic principle. If all faults behave in a similar way, we can use observations from different faults, each at different points of the cycle, to infer the time-dependent behaviour of an individual fault. This approach has been used to establish a few key patterns of behaviour that any successful model must be able to reproduce^11^,^100^. First, and with remarkably few exceptions, the deformation between earthquakes at major faults is focused around the fault (for example, Fig. 6), consistent with deformation in the lower crust occurring in relatively narrow shear zones beneath a locked elastic lid (for example, Fig. 7). Unfortunately, deformation data alone cannot distinguish between slip on a narrow fault plane and strain distributed across a shear zone; distributed strain in a shear zone up to three times as wide as the thickness of the seismogenic upper crust is indistinguishable from slip focused on a narrow fault^63^.

Second, the slip rates of major faults derived from simple steady-state geodetic models have been shown to agree within error with those estimated from dating geological offsets^100^,^101^. This suggests that strain rates are more or less steady for most of the earthquake cycle. This is surprising as a transient period of elevated strain is observed after most earthquakes (for example, Fig. 7). This postseismic deformation has been attributed to continued aseismic afterslip on the fault plane^102^, readjustment of groundwater following coseismic pressure changes (poroelastic deformation^103^), and viscous flow in the lower crust and/or upper mantle^104^,^105^. Measurable elevated strains occur for a few years to a few decades following earthquakes^11^. The agreement between slip rates from geology and geodesy suggests that postseismic deformation only affects a small fraction of the overall earthquake cycle. Nevertheless, the response of the crust in space and time following major coseismic stress changes provides the opportunity to probe the rheology of the crust and upper mantle. Investigations of postseismic deformation require that part of the system can respond quickly to these stresses, either through slip on the fault or viscous flow. In models where viscoleastic relaxation of a uniform Maxwell viscoelastic substrate is responsible for postseismic transients, relaxation times of ∼1 year (viscosities of ∼10^18^ Pa s) are typical.

There are a handful of locations distributed across the planet where we have good geodetic observations before and after an earthquake on a strike-slip fault (Fig. 7). In each of these cases, the strain rates observed in the immediate postseismic period are high, but they do not decay away completely by the end of the cycle, and we see a remarkably consistent pattern of focused interseismic strain late in the earthquake cycle. The results show that focused interseismic deformation and rapid postseismic deformation occur at the same location, and a single rheological model must be able to reproduce both signals.

Any successful model of the earthquake deformation cycle must therefore be able to reproduce both rapid postseismic transients and focused interseismic strain. Simple two-layer models with an elastic lid over a viscoelastic substrate^92^,^106^ can reproduce rapid postseismic deformation if the relaxation time of the substrate is short with respect to the earthquake repeat time (small *τ*_0_ curves in Fig. 7), or focused interseismic deformation late in the cycle if the relaxation time is long (large *τ*_0_ curves in Fig. 7), but not both. A number of alternatives have been suggested. A substrate with uniform bi- or tri-viscous rheology can reproduce the surface observations^107^. Alternatively, it has also been proposed^108^ that faults are underlain by a ‘weak zone' in an otherwise strong substrate; relaxation of the ‘weak zone' gives rapid postseismic deformation, whereas slow relaxation in the strong substrate is responsible for focused interseismic deformation (Fig. 7). Such a configuration is consistent with models based on rock mechanics^109^,^110^, where depth-dependent viscosity, power-law creep and shear heating can result in a region with lower effective viscosity at the base of the seismogenic layer, embedded in an otherwise strong lower crust and upper mantle. Further weakening may occur through grain size reduction^16^. In an alternative class of models, afterslip on a fault continuing beneath the seismogenic layer, controlled by rate-and-state friction^111^, has been shown to be capable of reproducing the surface observations at Parkfield section of the San Andreas fault^2^, without any requirement for viscoelastic relaxation.

## Outlook

**Figure 8:**
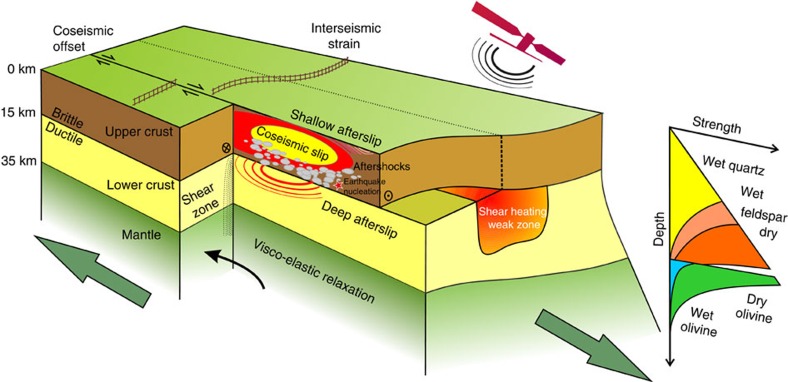
Conceptual cartoon of deformation in the crust and uppermost mantle. Satellite geodesy offers the opportunity to measure the complete earthquake cycle: first, coseismic slip in the seismogenic upper crust, its relationship with aftershocks and fault segmentation; second, postseismic deformation localized on fault structures as shallow and deep afterslip, or more widely distributed through the ductile lower crust and upper mantle flow as viscoelastic relaxation; and third, interseismic strain accumulation across fault zones between earthquakes. By using the high spatial and temporal resolution of satellite observations, it will become possible to determine the time-dependent rates of deformation as well as the spatial extent of shear zones and weak zones beneath faults. Improved measurements of these processes in time and space will allow us to better constrain the lateral variability and depth-dependent rheology within the crust.

The next decade should see us begin to discriminate between these models using more and better Earth Observation data that describe the evolution of deformation in space and time for an increasing number of earthquake faults (Fig. 8). The models make specific predictions about the temporal and spatial behaviour of deformation that can be discriminated with long time-series of observations. At the same time, complementary data from seismic imaging and rheological constraints from rock mechanics will be vital in solving this problem^16^.

On a broader scale, Earth Observation data are now reaching the spatial resolution and accuracy to enable us to assess the fundamental mechanics of how continents deform. We have known for decades that the continents do not deform as large rigid plates like the oceans^112^,^113^, but the kinematics and dynamics of continental deformation are still unclear^101^. The debate has historically been polarized between two end member views. In one, the continents have been considered to act like a viscous fluid, with internal buoyancy forces playing a key role in controlling the distribution of deformation, and faults only acting as passive markers reflecting the deformation of a deeper, controlling layer^114^. The alternative view has been that the continents can be considered to be a collection of rigid blocks, each behaving in essence like an independent plate^115^. Resolving this issue is important for earthquake hazard assessment–we need to understand the degree to which deformation and earthquakes are focused on the major, ‘block-bounding' faults, as opposed to being distributed throughout the continents. Long time-series of surface deformation data from Earth Observation satellites will enable us to quantify the degree to which deformation occurs away from the major ‘block-bounding' faults^77^.

An additional key question concerns the degree to which deformation observed in the upper crust occurs elastically. Geodetic models generally assume that the crust behaves elastically between earthquakes, yet geological structures often show plastic deformation. The degree to which the deformation we observe now at the surface reflects the accumulation of elastic strain on faults that will eventually be released in earthquakes, as opposed to plastic deformation that will built geological structures, remains unclear. Earth Observation data, in conjunction with careful field studies, has the potential to address this question. By combining long-term investigations of geological structures and their deformation rates with current deformation, we can assess the degree to which the strain currently accumulating around active fault structures is eventually released through slip on those structures.

We are entering an exciting new era of Earth Observation data. Satellite observations of fault structures and their rates of deformation are becoming available with high spatial and temporal resolution, and greatly improved accuracy. In particular, the coming decade will see a dramatic improvement in our ability to observe time-dependent phenomena, such as slow slip events^116^, and long-term transients, such as the accelerating deformation that was observed before the 2011 Tohoku earthquake^117^. By combining satellite observations on all spatial scales with new constraints from rock mechanics and seismic imaging, the next decade will require the development of new theories of active tectonics and earthquakes that should improve our ability to live safely on this hazardous planet.

## Additional information

**How to cite this article:** Elliott, J. R. *et al*. The role of space-based observation in understanding and responding to active tectonics and earthquakes. *Nat. Commun.* **7,** 13844 doi: 10.1038/ncomms13844 (2016).

**Publisher's note:** Springer Nature remains neutral with regard to jurisdictional claims in published maps and institutional affiliations.

## Earth Observing Systems

Active systems (radar): whilst the pioneering work done with orbital SAR was by the short Seasat mission in 1978, the first use of InSAR for tectonics was with the ESA European Remote Sensing satellite, ERS-1 (Fig. 2b), which measured the deformation due to the 1992 Landers (California) earthquake^5^. ESA's follow-up missions, ERS-2 and ENVISAT, provided near-global land coverage for deforming areas, although typical re-observation times were commonly many months rather than the shortest possible revisit frequency offered by the 35 day orbital return period. The three main wavelength bands in which most SAR imaging satellites operate are L, C and X-band, which have wavelenths of ∼20, ∼5 and ∼3 cm, respectively. The different wavelengths offer different strengths^129^. In comparison to C-band, L-band is better at maintaining interferometric coherence over vegetated areas, but suffers more from ionospheric path delays due to its lower frequency. X-band SARs currently in orbit operate in acquisition modes which acquire data at higher ground resolution but often in narrower swaths. The current SAR systems that acquire regular near-global land coverage are the Sentinel-1A/B (C-band) and ALOS-2 (L-band) satellites, which are well suited for measuring deformation at a continental scale. More targeted acquisitions are made by the higher resolution X-band satellites, COSMO-SkyMed and TerraSAR-X, but these acquire data typically covering smaller areas, making them more suited for city-scale studies rather than continental-scale deformation mapping, although they have proved useful for landslide, subsidence and volcano monitoring. Several future SAR constellations are planned, including systems of two (SAOCOM-1A/B and CSG-1/2) or three (RADARSAT Constellation Mission - RCM) simultaneous platforms in orbit; COSMO-Skymed already operates a four-satellite constellation and ESA operate two with Sentinel-1A/B.

Passive (optical/spectral): the earliest optical imaging satellites with global coverage were part of the NASA Landsat series, considered now to have relatively low 15–80 m resolutions. This pioneering series of satellites was initiated in 1972 with the launch of Landsat-1 and continues today with Landsat-8, launched in 2013 (Fig. 2a), providing a long-time series of land cover observations on a 16 day repeat basis^12^, and acquiring images in up to 11 spectral bands. Higher resolutions were achieved with the French SPOT series of satellites that have increased in resolving power through time from 10 to 20 m (SPOT 1–3) to 1.5–6 m today (SPOT 6/7). These platforms acquire data in a higher resolution panchromatic band as well as lower resolution red, green, blue and near-infra red channels. Sentinel-2A was recently launched and provides openly available data in 12 bands from visible through near to short wave infrared at 10–60 m resolution. The highest resolution images (<1 m) are currently the preserve of commercial satellite companies. IKONOS was the first to reach this resolution milestone in 1999; DigitalGlobe's series of Worldview satellites currently offers the highest resolution at 31 cm panchromatic on Worldview-3, with 16 spectral bands. The pair of Pleiades-1A/1B satellites offer flexible acquisition modes in 20 km wide swaths at 50 cm panchromatic resolution (2 m multispectral) and are capable of along-track stereo and tri-stereo, as well as wider area mosaics. Some past spy satellite images from the US Key Hole (KH) series in the 1960's and 70's (for example, Corona KH-4 ∼2 m/Hexagon KH-9 6-9 m), have in part been publicly released and can be used to look at cities that have since been built over active faults^130^, as well as to measure past deformation events such as earthquakes and rifting^48^.
